# Temporal trends in biopsy proven glomerular disease in Uruguay, 1990-2014

**DOI:** 10.1371/journal.pone.0206637

**Published:** 2018-10-29

**Authors:** Mariela Garau, Jimena Cabrera, Gabriela Ottati, Hena Caorsi, Francisco Gonzalez Martinez, Nelson Acosta, María Haydee Aunchayna, Liliana Gadola, Oscar Noboa

**Affiliations:** 1 Departamento de Métodos Cuantitativos, Facultad de Medicina, Universidad de la República, Montevideo, Uruguay; 2 Uruguayan Registry of Glomerulopathies, Montevideo, Uruguay; 3 Centro de Nefrología, Hospital de Clínicas, Facultad de Medicina, Universidad de la República, Montevideo, Uruguay; 4 Departamento de Anatomía Patológica, Hospital de Clínicas Facultad de Medicina, Universidad de la República, Montevideo, Uruguay; Istituto Di Ricerche Farmacologiche Mario Negri, ITALY

## Abstract

Our aim is to describe variations in the incidence rates of glomerular disease diagnosed by renal biopsies performed in Uruguay over the last 25 years in relation to sex, age, clinical presentation and histological diagnosis. We analyzed all renal biopsies performed in Uruguay during the 25 years period and estimated incidence rates per million people per year (pmp/yr) for the population older than 14 years. Mann Kendall's trend analysis was used to assess incidence trends. In order to identify changes in trends, we compared annual incidence rates with the Joinpoint method. From 1990 to 2014, 3390 biopsies of native kidneys corresponding to glomerular disease were performed in patients older than 14 years. The average biopsy rate was 58 per pmp/yr. The glomerular disease incidence rate increased progressively over the period (p<0.05). Trends analysis over five-year periods demonstrated a progressive increase of IgA nephropathy (3.08 pmp/yr 1990–1994 to 12.53 pmp/yr 2010–2014 p<0.05), membranous nephropathy (2.38 pmp/yr 1990–1994 to 8.04 pmp/yr 2010–2014 p< 0.05) and lupus nephritis (4,23 pmp/yr 1990–1994 to 7,81 pmp/yr 2010–2014 p<0.05). There was a change in the trend of focal segmental glomerular sclerosis (FSGS) which increased until 1996 and decreased afterwards. The incidence rates of glomerular disease have doubled globally in the last quarter of a century in Uruguay, mainly related to the increase of IgA nephropathy, membranous nephropathy and lupus nephritis. There was a change in the slope of the incidence rate of FSGS.

## Introduction

The incidence of glomerular diseases varies across geographic regions [1] and criteria for kidney biopsy has changed over time. Epidemiological information is scarce, particularly from Latin America, and rarely considers the information from all the country. A recent review of the incidence of glomerular diseases (GD) worldwide [2] showed only two articles from South American countries, with figures referring to the last decades of the twentieth century, one about pediatric population in Venezuela[3] and the other is the 2005 publication of the Uruguayan Registry of Glomerular diseases (URG) by Mazzuchi et al. [4].

Uruguay has a population of 3.3 million according to the national 2011 census [5]. The country has a homogeneous and stable population in terms of migratory behaviour.(95% Western European descent). Total population was 2.955.241 in 1985, 3.163.763 in 1996, 3.241.003 in 2004 and 3.286.314 in 2011.[6] According to the surveys held by the Uruguayan Ministry of Health using the STEPwise approach to Surveillance (STEPS) method in population aged 15–64, prevalence of hypertension was 30% both in 2006 and 2013, and prevalence of diabetes was 5.5% in 2006 and 6% in 2013. [7,8]

Renal replacement therapy (RRT) is accessible to the entire population due to a national fund that covers high cost procedures, such as chronic dialysis and kidney transplantation. In 2014, 68.5% of patients in RRT were on dialysis [758 patients per million people per year (pmp/yr)] and 31.5% had a functioning renal graft (340 pmp/yr). The incidence rate of GD as a cause of end stage renal disease was 15.7 pmp/yr, representing 10% of the patients who required RRT in 2014 [9]. In the period 1990–2014, considering all the patients that entered dialysis with diagnosis of GD (excluding diabetes nephropathy),forty one percent had clinical diagnosis without RB, 15% had focal and segmental glomerulosclerosis (FSGS), 8,5 vasculitis (VASC), 5% IgA nephropathy (IgAN), 5% lupus nephritis (LN) and 4,4% amyloidosis (AML) (S1 Table).

The URG was initiated by the Nephrology Department of the School of Medicine in 1970 as a hospital-based registry at the University Hospital (Hospital de Clínicas) and progressively included patients from other institutions. In 1989, it became a national population-based registry with data from all biopsied patients in the country, as described by Mazzuchi et al [4]. Glomerular disease report is mandatory since 2000. URG is a branch of the Programa de Prevención y Tratamiento de las Glomerulopatías (Glomerular Disease Prevention and Treatment Program)[10] that establishes National guidelines periodically updated, taking into account international guidelines as KDIGO[11].

We emphasize the importance of the information that arises from the registry to design diagnostic and therapeutic policies in the country and the region and contribute to understanding the causes of the changes in the incidence of glomerular diseases.

Our aim is to describe the variations in the incidence of GD diagnosed by renal biopsy in Uruguay over the last 25 years in relation to sex, age, clinical presentation and histological diagnosis.

## Material and methods

### Data source

The URG obtains data from all pathologists that inform native kidney biopsies in the country and also registers clinical data provided by treating nephrologists at the time of the renal biopsy (RB). In Uruguay, the RB is usually performed when a GD is suspected. During the first decades of the Registry, only GD diagnosis were recorded. Since 2000 we keep a record of all the diagnosis of renal biopsies, not only GD. The number of RB related to GD is almost 95% every year and the remaining 5% correspond to tubulointerstitial nephritis or acute tubular necrosis.Nephrologists perform all the renal biopsies. Since 1990 biopsies are performed with automatic needles guided by imaging, either tomography or ultrasound. Pathological study always includes optical microscopy and immunofluorescence. Electronic microscopy is performed when it is considered essential to complete the diagnosis.

The information is stored in two related databases, one for biopsy and initial clinical data and the other for the follow-up data. The first database includes the variables of identification of the patient: national identification number, sex, age and date of birth, in addition to the most relevant clinical and laboratory variables: biopsy date, time between onset of symptoms and time of biopsy, clinical presentation, blood pressure at the time of diagnosis, plasma creatinine, urine protein and presence of hematuria. Information about dialysis admission is verified or completed with the collaboration of the Uruguayan Dialysis Registry [9].

We retrospectively analyzed all GD in patients older than 14 years at the time of renal biopsy included in the URG from 1990 to 2014. In patients with more than one biopsy, only the first one was included for analysis. For the histopathological diagnosis, URG codes were grouped into the following categories: minimal change disease (MCD), FSGS, membranous nephropathy (MN), membranoproliferative glomerulonephritis (MPGN), IgAN including Schönlein Henoch syndrome, non-IgA proliferative mesangial glomerulonephritis (MGN), post infectious glomerulonephritis (PIGN), chronic sclerosing glomerulonephritis (CSGN), LN, diabetic nephropathy (DIABN), VASC, AML, monoclonal immune deposition disease (MIDD) and other glomerular diseases associated with systemic disease. Both MN and FSGS included primary and secondary glomerular diseases, since there were no clinical data available to distinguish between them, except for lupus MN which was included in the LN category. C3 Glomerulonephritis and dense deposits disease were included in MPGN. Pauci-immune glomerulonephritis, either systemic or limited to the kidney were included in the vasculitis category. In some cases, the pathologist did not find any glomerular lesion related with proteinuria he but did find intratubular red cells confirming glomerular origin of hematuria, those cases were coded non classifiable glomerular disease (NCGD) in absence of electron microscopy.

We classified clinical presentation into one of eight categories: nephrotic syndrome (NoS) (urine proteinuria > 3.5 g/day, albuminemia <3 g/dL and dyslipidemia), nephritic syndrome (NiS) (glomerular hematuria in the presence of renal dysfunction defined by non-nephrotic proteinuria and/or microhematuria with hypertension and/or high plasma creatinine, macroscopic hematuria (MH), hypertension (HT), acute kidney injury (AKI), asymptomatic urinary abnormalities (AUA), chronic kidney disease (CKD), and rapidly progressive glomerulonephritis (RPGN) (doubling of creatinine or decreased glomerular filtration rate by half in three months associated with urine sediment abnormalities). Hypertension was defined as an office blood pressure of at least 140 Hg mm systolic or 90 Hg mm diastolic or receiving antihypertensive drugs. AUA was defined as changes in the urinary sediment (proteinuria > 0.5 gr/24hrs, hematuria, urinary casts), without hypertension, and with an estimated glomerular filtration rate > 60 ml/min/1.73 m^2^ estimated by the Chronic Kidney Disease Epidemiology Collaboration (CKD-EPI) equation.

Age at the time of renal biopsy was grouped for analysis purpose as follows: 15–35, 36–50, 51–65, >65 years.

### Statistical analysis

The results are presented as mean and standard deviation (SD) for the continuous variables; qualitative variables are described with percentages.

We estimated incidence rates per million people per year for the country’s population older than 14 years by linear interpolation of census data from 1985, 1996, 2004 and 2011. [5,6,12]. In order to lessen the variability derived from the small number of observations, the analysis was performed by grouping data into 5-year time frames: 1990–1994, 1995–1999, 2000–2004, 2005–2009, and 2010–2014. Mann Kendall's test was used to assess incidence trends over the period. However, this test only identifies monotonic trends and does not detect changes in trends over time. Therefore, to identify changes in trends we compared annual incidence rates with the Joinpoint method [13,14] in those diagnostic groups where the number of cases was large enough.[15] This method uses a model in which the logarithm of the incidence rate is a linear function of time and it is possible to assess the annual percentage change (APC) to quantify the magnitude of the change in incidence rate.

We used a chi-square test to investigate the association of categorical variables. A p-value < 0.05 was considered significant. The analysis was performed with the statistical package SPSS (Statistical Package for Social Sciences Inc. Chicago, Illinois, USA) version 15.0 for Windows. [16]

### Ethical aspects

The protocol for the analysis to be performed was accepted by the ethics committee of the Hospital de Clínicas from the University of the Republic. Strict confidentiality was assured in all cases.

Glomerular disease notification to the URG is mandatory by Health Secretary ordinance (ord 324/2000). Informed consent was obtained for epidemiological research at the moment of RB or when patient entered renal health program. The ethics committee authorized the review of clinical data of those patients who died without signing informed consent.

## Results

### Demographic data

From 1990–2014, 3390 biopsies of native kidneys corresponding to GD were performed in patients older than 14 years. The mean annual incidence of GD was 58 per million people per year (pmp/yr).

Patient mean age was 43 ± 18 (range 15 to 92) years. Over the 25 years period, the age distribution changed; the proportion of individuals aged less than 35 years decreased and patients over 65 years increased. Mean age showed an upward trend over time (p <0.05) (Table 1).

**Table 1:** Temporal trends in patient demographics among the study cohort of glomerular diseases confirmed by renal biopsy by sex and age category in Uruguay.

	1990–1994 n = 443	1995–1999 n = 613	2000–2004 n = 673	2005–2009 n = 795	2010–2014 n = 866
**Age Mean (SD)**	41.1 (17.5)	42.3 (17.7)	42.6 (18.4)	44.9 (18.6)	44.1 (18.3)
**Age category**					
**15–35**	36.3%	38.3%	38.0%	31.9%	34.6%
**36–50**	24.6%	23.5%	25.0%	23.0%	23.4%
**51–65**	20.8%	21.0%	16.9%	21.9%	20.6%
**>65**	9.0%	11.7%	16.5%	16.2%	16.5%
**Missing Age**	9.3%	5.4%	3.6%	6.9%	4.8%
**Men**	51.0%	54.5%	52.7%	54.6%	50.7%

**Fig 1:**
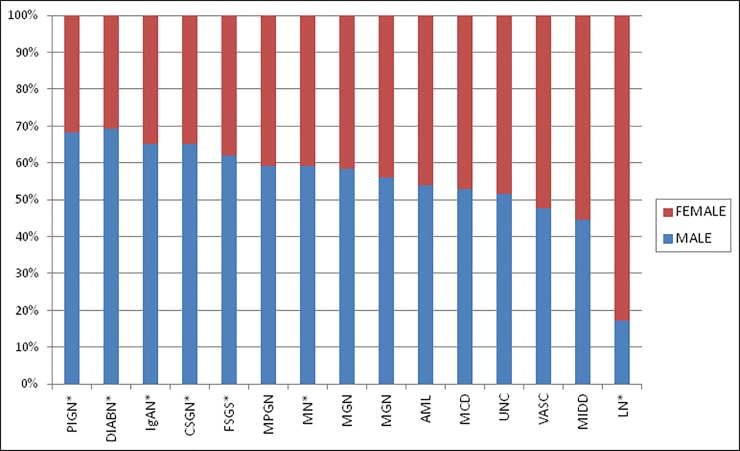
Sex distribution by diagnosis. PIGN Postinfeccious glomerulonephritis; DIABN Diabetic nephropathy; IgAN IgA nephropathy; CSGN Chronic Sclerosing glomerulonephritis; FSGS Focal Segmental Glomerulosclerosis, MPGN Membranoproliferative glomerulonephritis; MN Membranous nephropathy; MGN Mesangial glomerulonephritis (Not IgA); AML Amyloidosis; MCD Minimal Change disease; UNC Unclassifiable; VASC Vasculitis; MIDD Monoclonal immune deposition disease; LN Lupus nephritis. * Significantly different from 50% (p<0.05).

Over the whole period, the male sex slightly predominated (52.7%) (Table 1). Primary GD were diagnosed predominantly in men (59.6%); while secondary ones were diagnosed mainly in women (61.3%) (Chi^2^: p <0.001), with LN showing a female-biased sex ratio of 4.5:1. Fig 1 shows the sex distribution according to diagnosis.

### Diagnosis: Incidence and trends

The biopsy proven GD rate increased progressively over the period (Mann Kendall test, p <0.001). Table 2 shows the variations in GD incidence rate in the different age groups. There was an increase in the GD incidence rate in all age groups (Mann Kendall test, p <0.05) with the exception of the 59–64 age group. The largest increase was observed in the group > 64 years, among whom the glomerulopathy incidence rate was 2.33 times higher from 2010–2014 than from 1990–1994.

**Table 2:** Glomerular disease incidence rate per million person-year by sex and age category in Uruguay. 1990–2014.

	1990–1994 n = 443	1995–1999 n = 613	2000–2004 n = 673	2005–2009 n = 795	2010–2014 n = 866	Mann Kendall test (p value)
**Age category**						
**15–35**	35.3	48.8	53.6	52.6	62.2	0.043
**36–50**	39.2	48.8	55.2	59.7	64.7	0.014
**51–65**	40.5	57.0	49.3	71.1	67.7	0.11
**>65**	21.2	35.3	52.1	57.8	61.1	0.014
**Sex**						
**Men**	41.2	58.9	61.2	73.2	72.1	0.043
**Female**	36.2	44.8	49.6	54.7	62.4	0.014
**Total**	39.0	52.2	55.3	63.3	67.0	<0.001

Considering the twenty five years period, FSGS was the most frequent primary GD, followed by IgA nephropathy and membranous nephropathy. In 1057 cases, the GD was secondary to a systemic disease. The most frequent diagnosis was vasculitis, followed by lupus nephritis, amyloidosis and diabetes. In 11% of the biopsies it was not possible to conclude a cause of glomerulopathy. The specific incidence rates for each GD are shown in Table 3.

**Table 3:** Temporal trends in glomerular disease incidence rate per million person-year by diagnosis. Five year periods. Uruguay. 1990–2014.

	1990–1994	1995–1999	2000–2004	2005–2009	2010–2014	Mann Kendall test (p value)
**Primary GD**						
Minimal change disease	2.99	5.11	3.95	3.43	4.41	0.40
Focal segmental glomerulosclerosis	8.28	12.01	9.37	6.93	7.66	0.77
Membranous Nephropathy	2.38	3.83	5.51	5.97	8.04	0.014*
Membranoproliferative GN	0.62	1.19	1.64	1.91	2.17	0.014*
IgA Nephropathy	3.08	5.45	8.14	12.11	12.53	0.014*
Proliferative Mesangial GN (not-IgA)	0.88	0.51	0.00	0.88	0.54	**
Post infectious GN	0.88	1.45	1.32	0.64	0.69	**
Chronic sclerosing GN	0.70	1.62	0.74	1.35	0.54	**
Primary. Others	0.62	0.85	0.49	0.32	0.31	**
**Secondary GD**						
Lupus nephritis	4.23	4.68	5.76	7.01	7.81	0.014*
Diabetic nephropathy	0.09	0.43	0.99	0.80	1.62	0.04*
Vasculitis	5.55	5.88	7.89	8.76	7.81	0.11
Amyloidosis	0.88	0.94	0.90	2.55	1.78	0.11
MIDD	0.09	0.26	0.99	1.27	1.16	0.04 *
Secondary.Others	0.44	0.77	1.56	1.83	1.31	**
**Non classifiable GD**	6.78	7.24	4.77	6.13	7.19	0.50
**Total**	38.48	52.19	54.03	61.89	65.57	p<0.001

GD Glomerular disease GN Glomerulonephritis MIDD Monoclonal immune deposition disease

(*) monotonous tendency. p<0.05

**Not tested. NS Not significant

Trends analysis for five-year periods demonstrated a progressive increase of IgAN (3.08 pmp/yr 1990–1994 to 12.53 pmp/yr 2010–2014 p <0.05), as well as of MN (2.38 pmp/yr 1990–1994 to 8.04 pmp/yr 2010–2014 p < 0.05), and of MPGN (0.62 pmp/yr 1990–1994 to 2.17 pmp/yr 2010–2014 p <0.05). The incidence of LN also increased significantly (4.23 pmp/yr 1990–1994 to 7.81 pmp/yr 2010–2014 p <0.05).

Although the FSGS incidence rate did not show a significant monotonous trend, the Joinpoint analysis using the annual incidence rates showed a progressive increase until 1996, where it reached a rate of 12.2 pmp/yr, followed by a decrease with an APC of -3.49%, significantly different from 0 (p < 0.001). As a result of this decrease and the concomitant increase in the incidence of other glomerular diseases, IgA nephropathy became the GD with the highest incidence rate in the last two periods (12.11 and 12.53 cases pmp/yr, respectively). In the same two period FSGS incidence rate was 6.93 and 7.66 cases pmp/yr, respectively. In the 2010–2014 period, membranous nephropathy had the second highest incidence with 8.04 cases pmp/yr. Vasculitis and LN share the first place with the higher incidence of GD related to systemic disease.

**Fig 2:**
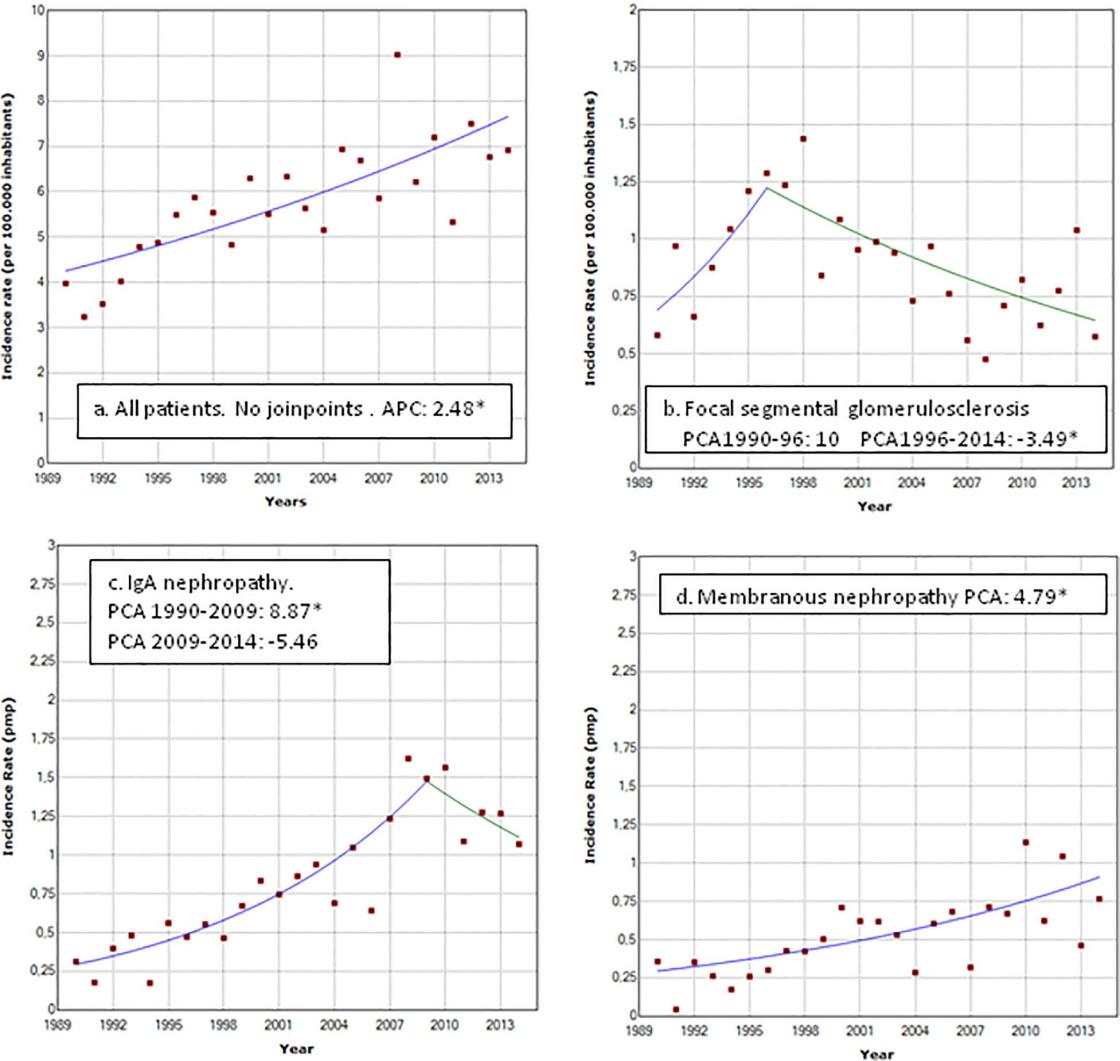
Trends in incidence rate of renal biopsy. **Uruguay. 1990–2014**. a. All patients b. FSGS c. IgA nephropathy d. Membranous nephropathy. * Annual percentage change statistically different from 0. (p<0.05).

Fig 2 shows the regression curve and the respective APC obtained with the Joinpoint analysis for all data and for the most frequent glomerular diseases diagnosed.

### Clinical presentation

**Fig 3:**
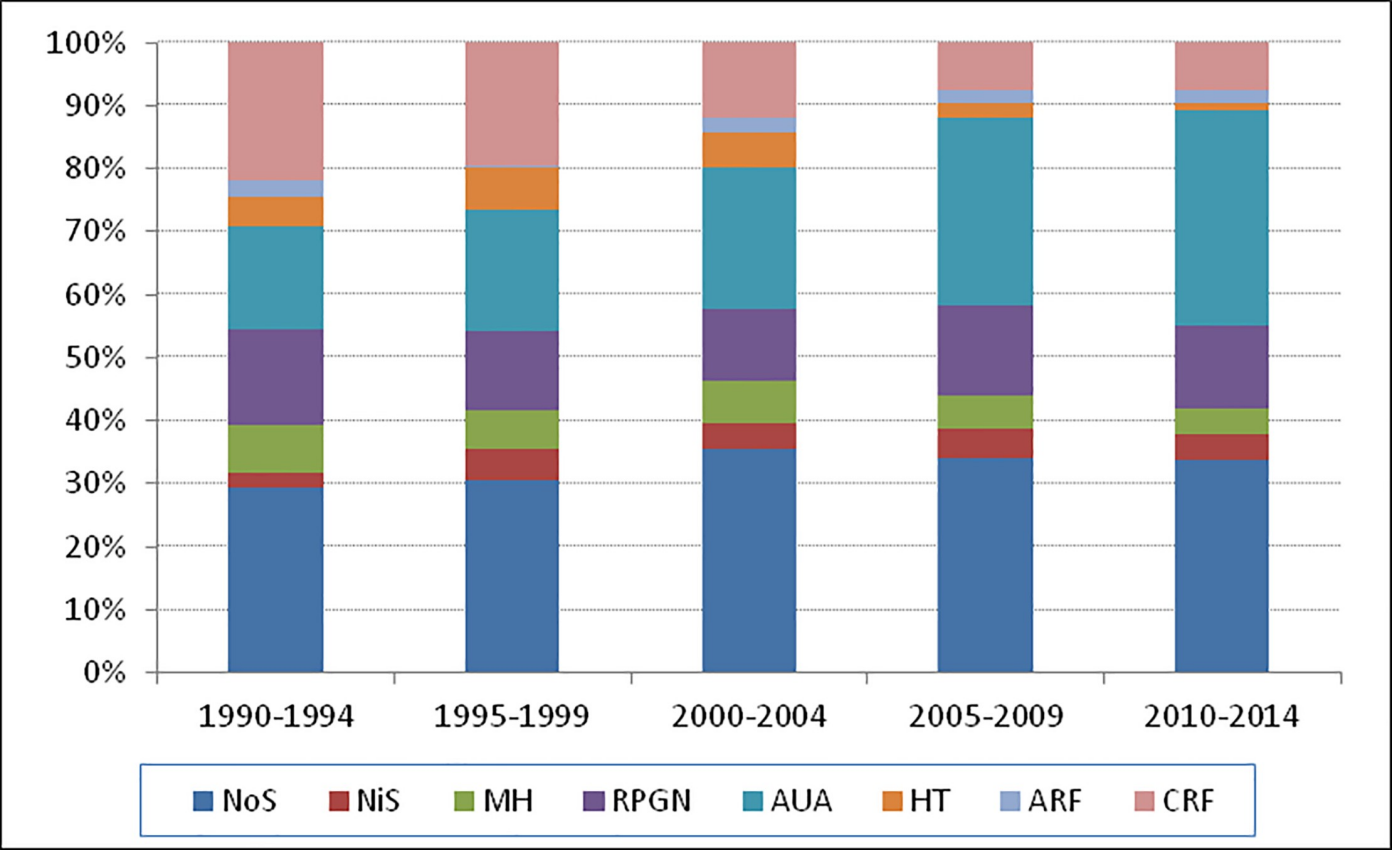
Clinical presentation by era. **Uruguay 1990–2014.** 1990-2014.NoS Nephrotic syndrome; NiS Nephritic syndrome; MH Macroscopic hematuria; RPGN rapidly progressive Glomerulonephritis; AUA urinary asymptomatic abnormalities; HT Hypertension; AKI Acute kidney injury; CKD Chronic kidney disease.

Clinical presentation data were available in 62% of the GD cases. From 1990–2014, the most frequent indication for renal biopsy was NoS (20.6%), followed by AUA (15.8%), RPGN (8%), CKD (8%), and macroscopic hematuria (3.6%). Although NoS was the first indication for RB in the overall data set, AUA was an increasingly prevalent indication, ranking first in 2010–14 (Fig 3).

**Fig 4:**
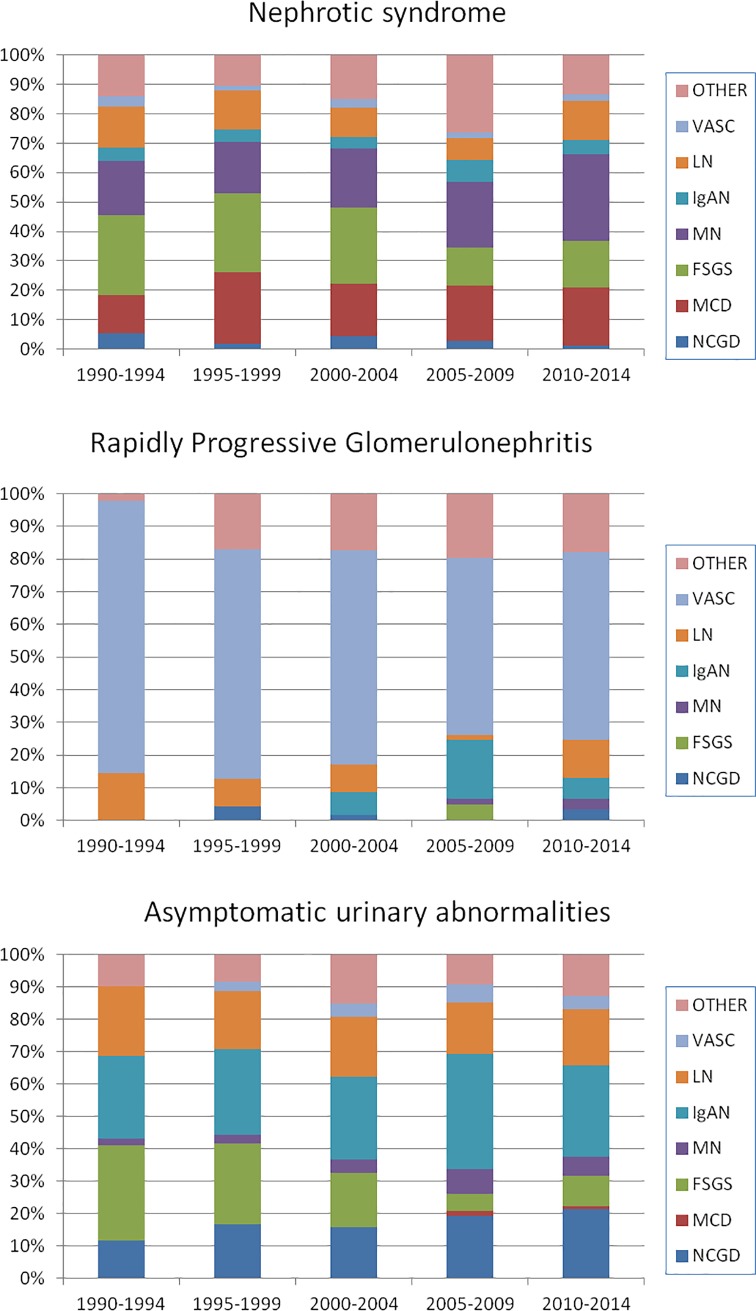
Diagnosis by eraby category of clinical presentation. **Uruguay. 1990–2014.** NCGD Non classifiable Glomerular Disease, MCD Minimal Change disease, FSGS Focal Segmental Glomerulosclerosis, MN Membranous nephropathy, IgAN IgA nephropathy; LN Lupus nephritis, VASC Vasculitis.

When NoS was the indication for RB, 22.2% corresponded to membranous nephropathy, 21.1% to FSGS, 18.8% to MCD and 11% to LN. Among those with AUA as indication of RB, the most frequent diagnosis was IgA nephropathy (28.8%), lupus nephropathy (17.8%) and FSGS (14%). In 18.2% of the patients who were biopsied because of AUA, a specific GD diagnosis could not be reached, although intratubular red cell casts were found, confirming a diagnosis of glomerular hematuria. Unfortunately electron microscopy was only performed in some of these cases. Fig 4 shows how the frequency of each diagnosis changes over time in the three more frequent categories of clinical presentation (NoS, RPGN, AUA). There was a relative decrease of FSGS diagnosis with an increase of MGD among patients with nephrotic syndrome. In patients with AUA, there was also a relative decrease of FSGS and a progressive increase of IgAN.

### Histological diagnosis related to age of presentation

**Fig 5:**
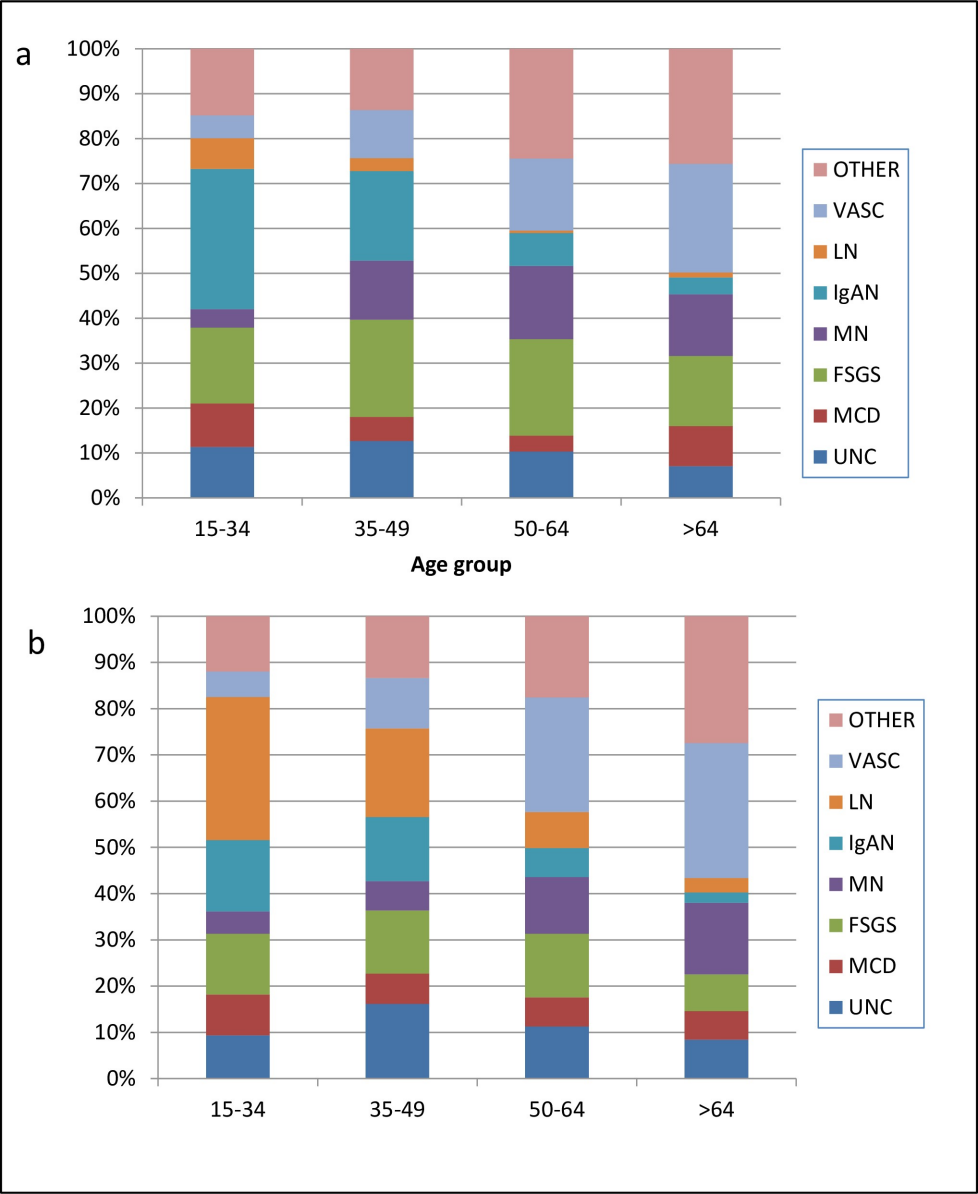
Diagnosis by age group. **Uruguay 1990–2014**. a. Males b. Females NCGD Non classifiable Glomerular Disease MCD Minimal Change disease FSGS Focal Segmental Glomerulosclerosis, MGN Membranous GN; IgAN IgA nephropathy; LN Lupus nephritis; VASC Vasculitis.

When all the data from 1990 to 2014 were analyzed, the most frequent diagnoses were: a) IgA nephropathy (23.7%), lupus nephritis (18.3%) and FSGS (15.3%) in the 15–35 year age group; b) FSGS (18.3%) and then IgAN (16.8%) in the 36–50 year age group, c) vasculitis (27.1%), FSGS (18.2%) and membranous GD (14.3%) in the 51–65 year age group, and d) vasculitis (27.1%), followed by membranous GD (15.9%) and FSGS (11.9%) in patients > 65 years. (Fig 5).

**Fig 6:**
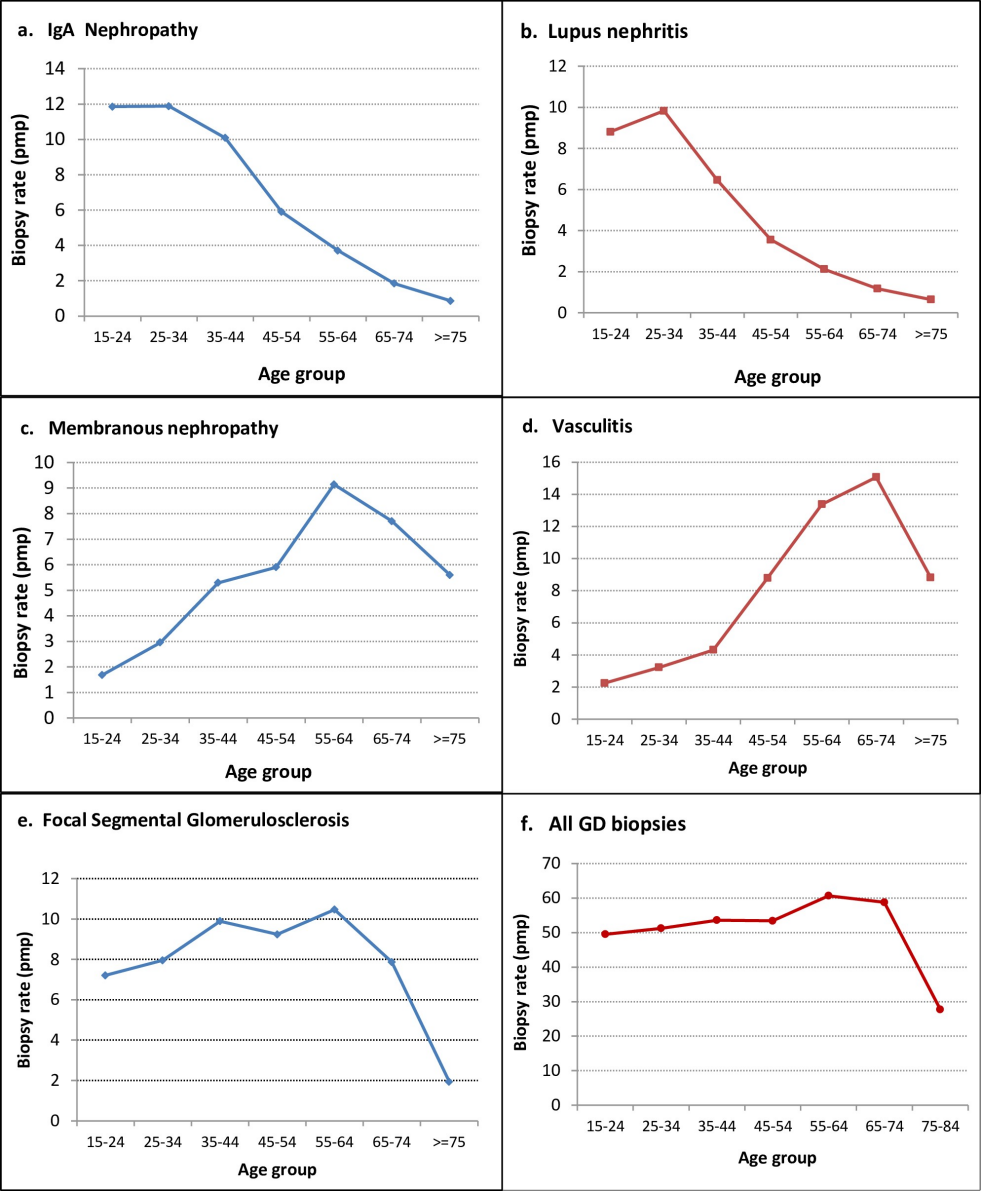
GD biopsy age specific incidence rates. **Uruguay. 1990–2014**. a. IgA nephropathy b. Lupus nephropathy c. Membranous nephropathy d. Vasculitis e. Focal segmental glomerulosclerosis e. All biopsies.

The biopsy rates in each age group under 75 years are similar, between 50 and 60 pmp/yr (Fig 6F) with lower values for the group older than 75. However, when analyzing the different diagnose, IgAN and lupus had high rates in the first decades of life, decreasing almost to 0 in subsequent decades (Fig 6A and 6B). On the other hand, membranous nephropathy and vasculitis rates progressively increased with age (Fig 6C and 6D). The rates were similar for FSGS through different age groups decreasing for patients older than 75(Fig 6E).

**Fig 7:**
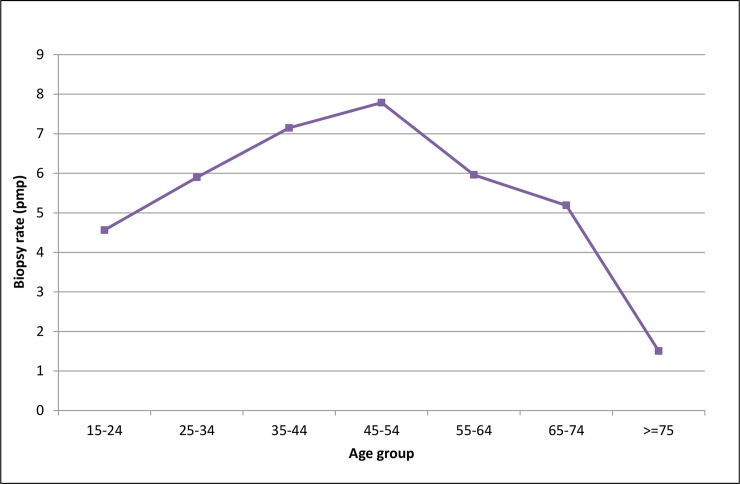
Age specific incidence rates. Non classifiable GD. Uruguay. 1990–2014.

Among patients with NCGD rates rise with age, peak at the 45–55 group and drop for the subsequent age groups (Fig 7).

#### Glomerular diseases in patients > 65 years

The incidence rate of GD in people > 65 years of age increased significantly. In this age group, the most frequent diagnoses were vasculitis and MN, followed by FSGS. The number of biopsied patients aged 80 years and older increased progressively (6 in 1990–1994, 5 in 1995–1999, 8 in 2000–2004, 15 in 2005–2008 and 17 in 2010–2014). In this group 57% were female while female comprise 67% of the population aged 80 years and older.

## Discussion

We performed a retrospective study of all the native kidney biopsies with diagnosis of glomerular disease in Uruguay in a 25 years period between 1990 and 2014.

The main strength of this work is that we were able to report the incidence rates of biopsy proven GD and their variation over time. This was possible because the URG is a nationwide registry that includes all renal biopsies in Uruguay and the size of the referral population is retrieved from Uruguayan periodical census. There are very few studies that analyze the epidemiology of glomerular diseases and their variation overtime with a population base. These studies include: The Olmsted County, USA study, that analyzes 375 renal biopsies in a thirty year period [17], a 30—year report of biopsies in a region of the United Kingdom [18], a Czech national study [19,20], a North German study with 222 biopsied patients [21], the Spanish registry [22] and the area of Cötes d'Armor, France[23]. Most published studies, report relative frequencies of GP diagnosed by biopsy, not providing information about incidence rates nor variations of the incidence rates of each glomerulopathy [24–27].

### Demographic and clinical presentation

The number and rate of renal biopsies corresponding to glomerular diseases in patients older than 14 years in Uruguay have increased progressively. During the 25 –year period the rate of RB corresponding to GD increased by 72% from 38.5 pmp/yr (1990–1994) to 65.6 pmp/yr in 2010–2014. This increase is similar to that found in other population-based registries such as Olmsted County, Minnesota [17]. These rates are not only related to the actual incidence of the different GDs, but also to the nephrologist’s biopsy criteria. In the last year group analyzed, Uruguay had an intermediate incidence rate of biopsied GD among the national registries from which data were available, (Serbia: 10 pmp/yr; Australia: 215 pmp/yr) [28]. This increase is observed in all age groups but is remarkable in patients older than 64, in which the incidence rate reached 56.1 pmp/yr (2010–2014). The rate increase for this group was 2.3 times higher than in 1990–1994 (Table 2).

Glomerular diseases were more frequent in men, except for LN, MIDD and vasculitis, which were more frequent in females (Fig 2). The most common clinical presentation during the whole period was nephrotic syndrome. However, in the last five years, AUA was the first indication for RB, a fact that probably is related to a change in nephrologists’ attitude, that are now more likely to biopsy earlier, in order to attain timely diagnosis.

### Temporal trends in glomerular disease incidence

The incidence rates of the different GDs has changed over the 25—year period analyzed. From 1990–1994 FSGS was the GD with the highest incidence, in agreement with published data from other South American countries [29,30]. In the last two periods, 2005–2009 and 2010–2014 IgAN was the most frequent diagnosis, in agreement with data from many developed countries such asItaly [31,32], Spain [22], Australia [33], Japan [34,35], and Korea [24].

The temporal trend of FSGS incidence rate changed during the 25 years period analyzed. Although this change was not demonstrated by the Mann Kendall test, the Joinpoint model shows a break point in 1996. There was an increase in the incidence rate between 1990 and 1996 that was not significant, probably due to an insufficient number of points, and afterwards there was a significant decrease since 1996. A possible explanation suggests a real change in the frequency of the diagnosis of FSGS, although there may be other interactions related to the indication of the RB. In the Olmsted registry the incidence of FSGS increased almost 13 times between the periods 1974–1983 and 1994-2003(P <0.001)[17]. There is no information if there was a decrease after that date. On the other hand, European reports showed low incidence, 1.5 pmp/yr cases of FSGS with no changes in time [18].

In this report IgA nephropathy is mainly diagnosed in young adults in accordance with other international reports [2,36]. The mean age of the patients was 33.8 years, and it was the most frequent diagnosis in the group aged 15–35 years. A diagnosis of IgAN is more common in men than in women, except for the Arizona registry [27]. The rate of IgA nephropathy increased significantly from 3.1 pmp/yr to 12.5 pmp/yr, concomitantly with an increase in the number of biopsies associated with AUA. It is possible that this simultaneous increase is due not only to an increase in the actual incidence but to an extended practice of indicating RB in patients with AUA. The Joinpoint analysis shows that the increase in the rate of IgA nephropathy reached a maximum in 2009, maintaining stable figures during the last five years, as observed in other diagnosis when procedures for early detection of a disease are developed. It should be noticed that the population analyzed is predominantly white, with European ancestry, which explains the high frequency of IgA nephropathy. Registries with a high percentage of black population show a low frequency of IgAN [25].

The incidence rate of membranous nephropathy increased significantly from 2.38 to 8.04 pmp/yr during the 25-year period. A similar increase in the incidence rate of membranous nephropathy is reported by Hanko et al. in the UK (3.9 to 10.5 pmp/yr)[18].

When analyzing secondary glomerular diseases, we observed an increase in the incidence rate of lupus nephritis ranging from 4.23 to 7.81 pmp/yr (p < 0.014) throughout the period, probably linked to better diagnostic methods of lupus and to an early approach to diagnosis by renal biopsy. The current incidence of 7.81 pmp/yr is similar to that reported by other registries (Table 4).

**Table 4:** Reported glomerular disease incidence rate (pmp/yr).

	Country	Years	Renal Biopsy	GD	IgAN	FSGS	MCD	MN	LN
Swaminathan S et al. * ^a^ [15]	Olmsted, MN, USA	1974–2003	**82–175**	**39–90**	7–21	1–18	2	4–10	1–0.7
Hanko JB et al^.^[16]	UK	1976–2005	**20.2–70.8**	**10.8–42.0**	3.4–17.9	0.6–1.8	1–4.6	3.9 - 10.9	
Sim JJ et al.[23]	CA, USA	2000–2011		**53–107**	1–16	16–53	12–4	7–14	
Maixnerova D et al.*[18]	Czech Rep	1994–2011	**58**	**50**	11.6	3.9	3.4	4	
Braun N et al.[19]	Northern Germany	2003–2008	**65.5**		20.7	11.2	3.2	5.2	
Rivera F et al.*[20]	Spain	1994–1999	**48**		7.9	6.4	4.8	6.2	5.6
Briganti EM et al.*[31]	Australia	1995–1997	**215**	**126**	42.9	21.2	5.6	13.3	17.4
Simon P et al[21]	Côte d'Armor, France	1976–2002		**77**	28	8		22	

*Pediatric population included.

a: Adjusted incidence rate for USA population year 2000

b.Primary GD only

In our country diabetic patients are biopsied only when non diabetic glomerulopathy is suspected. Indications for RB in diabetic patients are: persistent microhematuria or atypical features in the absence of target organ damage related to diabetes.Renal biopsy in diabetic patients was mainly performed in the absence of diabetic retinopathy or when the clinical presentation was atypical. This clinical approach explains why the percentage of biopsies corresponding to diabetic nephropathy was very low when compared to other registries [26]. As far as we do not register comorbid conditions we cannot inform the percentage of patients with diabetes that were biopsied.

The differences observed in the incidence of GD among studies may be related to geographical heterogeneity. Variations observed in this study cannot be attributed to geographical differences, but can be related to changes in environmental, lifestyle, or practice factors. Our report underlines these changes, although prospective studies are needed to fully understand the mechanisms involved.

The limitations of this study are those related to a registry-based study. As long as only those patients with persistent proteinuria are biopsied, the rates of biopsy proven renal disease underestimate the true incidence of the disease. The figures reported probably reflect the minimum incidence of moderate or severe disease.

It is difficult to discern whether incidence changes are related to real changes in the occurrence of different glomerular diseases or are they related to changes in renal biopsy indication. Undoubtedly there is a certain degree of ascertainment bias that may be related to nephrologists´ proclivity to perform renal biopsies. The improvement of biopsy needles and the incorporation of echographic guided biopsy, reduced biopsy risk. Furthermore, the threshold proteinuria for biopsy has decreased from persistently higher than 1 g/day, to 0.5 g/day in the last decades. This may have particularly contributed to the increase in biopsies due to asymptomatic urinary abnormalities.

On the other hand several aspects may have contributed to maintain a homogeneous attitude regarding renal biopsy throughout the country: a National Glomerulopathy Registry since 1989, mandatory glomerular disease report since 2000, National guidelines periodically updated [37] and regular sessions for case discussion.

In conclusion, ascertainment bias cannot be excluded but incidence rate changes along time probably also reflect real incidence rate changes of GD in the country. Nevertheless, this information may be relevant for public health awareness and planning, and contribute to understanding regional differences in the incidence of glomerular diseases. It is hoped that improved accuracy of diagnosis would improve patient outcomes due to more appropriate treatment. However this increase in diagnosis would have service planning implications. National health services need adequate resources to provide immunosuppressive treatments e.g. day wards for infusion treatments and funds for high cost immunosuppressive medications. It could be noted that long term healthcare costs might ultimately be decreased, if appropriate treatment of GD reduced progression to ESRD and need for RRT.

## Conclusions

A Nationwide Glomerulopathy Registry is useful for analyzing the evolution of GD incidence rates. These rates have doubled globally in the last quarter of a century in Uruguay, mainly related to the increase of IgAN and MN, with a change in slope of the incidence of FSGS, which increased until 1996 and afterwards showed a decrease in its incidence rate. We also observed a significant increase in the incidence rate of lupus nephritis. Most published studies, report relative frequencies of GP diagnosed by biopsy, not providing information about incidence rates of each glomerulopathy.

These data may be useful for clinicians evaluating patients with GD and for researchers planning to include different groups of patients in prospective studies.

Renal biopsy rates increased in patients over 65and biopsies were performed in patients older than 80. This change in clinical practice increased the diagnosis of glomerular diseases in these age groups that require specific treatment.

## Supporting information

S1 Table Number of cases of patients entering dialysis treatment as a consequence of glomerular disease per year. Uruguay. 1990–2014.
(XLSX)

S2 Table Number of gd cases by diagnosis stratified by age groups and gender over time. (XLSX)

S3 Table Microdata. (XLS)

S4 Table Codes. for microdata. (XLSX)
